# NCATS TL1/T32 William Schnaper Visiting Scientist program: Trainee-driven mini-symposium

**DOI:** 10.1017/cts.2026.10802

**Published:** 2026-07-21

**Authors:** Alexander Thomas Brunfeldt, Brittany M. Hufft-Martinez, Jason S. Hatfield, My Linh H. Nguyen-Novotny, Kathryn Sandberg, Dexter Lee, Desiree M. Sigala

**Affiliations:** 1 Wright Center for Clinical and Translational Research, Virginia Commonwealth University, Richmond, VA, USA; 2 Cell Biology and Physiology, The University of Kansas Medical Center, Kansas City, KS, USA; 3 Veterinary Population Medicine, University of Minnesota, St. Paul, MN, USA; 4 Clinical Translational Science Center, Weill Cornell Medicine, New York, NY, USA; 5 Georgetown-Howard Universities Center for Clinical and Translational Science, Georgetown University, Washington, DC, USA; 6 Georgetown-Howard Universities Center for Clinical and Translational Science, Howard University, Washington, DC, USA; 7 Clinical and Translational Science Center (CTSC), University of California Davis, Sacramento, CA, USA

**Keywords:** Mini-symposium, predoctoral student, postdoctoral fellow, NCATS, clinical translational science

## Abstract

The William Schnaper Visiting Scientist (WSVS) Mini-Symposium was established within the National Center for Advancing Translational Sciences (NCATS) Clinical and Translational Science Award (CTSA) consortium to provide predoctoral and postdoctoral trainees with opportunities to present research, develop communication skills, and foster cross-institutional collaboration through a fully virtual, trainee-centered format. We describe the structure, implementation, and outcomes of seven WSVS Mini-Symposia conducted from 2021 to 2025. Program metrics included participation across CTSA hubs, trainee roles, research domains represented, and survey-based evaluations from presenters and attendees assessing perceived value, professional development, and program logistics. Seven symposia featured 62 trainee presenters from 28 CTSA hubs across 21 U.S. states. Most presenters were predoctoral trainees (79%), and research spanned basic (34%), clinical (52%), and implementation/public health (13%) domains. Survey responses indicated strong perceived benefit: 100% of presenters reported value for professional development and roughly 50% reported receiving useful feedback and establishing new professional contacts; 78% of attendees reported the symposium was a valuable learning experience. The WSVS Mini-Symposium demonstrates that a scalable, trainee-led virtual forum can effectively promote scientific communication, networking, and workforce development across geographically distributed training programs. This model provides a sustainable framework for enhancing translational science training nationwide.

## Introduction

With the growing demand for innovative medical treatments, biomedical science has become a rapidly expanding field fueled by technological advancements, scientific discovery, and evolving health care delivery. To address the growing need for innovation and collaboration in biomedical research, the National Institutes of Health (NIH) established the Clinical and Translational Science Award (CTSA) program in 2005 to create multidisciplinary and integrated centers for clinical and translational science at leading academic medical institutions [1]. These centers serve as hubs for translational research, providing the infrastructure, resources and expertise needed to accelerate discoveries. Since 2011, the CTSA program has been administered through the National Center for Advancing Translational Science (NCATS), whose mission is to leverage translational science to more rapidly transform research findings into health solutions [2].

In alignment with the CTSA program’s mission to advance clinical and translational science through education and training, the CTSA-linked training award (TL1/T32) program was established to provide research and workforce development opportunities for pre- and post-doctoral trainees [1]. Core components of the TL1/T32 programs include delivering foundational knowledge and practical skills in translational science, as well as promoting the training of scientists from diverse backgrounds to effectively bridge basic, clinical, and population research. As of August 2025, 41 institutions hold active CTSA awards, supporting 47 TL1/T32 programs across the United States [3].

As translational research landscapes become increasingly complex, training programs must evolve to equip the next generation of scientists with the skills and experiences necessary to succeed across disciplines and sectors. Inspired by and adapted from the NCATS K12 Mentored Research Career Development Program Award, which piloted visiting scientist exchanges among CTSA hubs [4], the William Schnaper Visiting Scientists (WSVS) program was conceived in 2018 during an NCATS CTSA Program meeting. Designed as a scalable, inclusive initiative, the WSVS program aimed to expose predoctoral and postdoctoral TL1/T32 trainees to diverse career paths, facilitate cross-institutional mentorship, and promote national scientific exchange through three fully virtual venues: the Debate Forum, Grand Rounds Series, and Mini-Symposium. The Debate Forum has two teams of trainees debate either side of a controversial topic in clinical and translational science; for example, the Fall 2022 debate titled “Can we trust medical guidelines” had trainees debate the applicability and feasibility of medical guidelines in diagnosing and treating patients. The Grand Round Series pairs one trainee with a partner institution, where he or she gives a 1-hour research talk, has 3 to 4 one-on-one meetings with faculty, and has a virtual lunch with other trainees. The mini-symposium, which is the focus of this manuscript, empanels a group of trainees to present an 8-minute research talk across the 47 CTSA programs, nationwide. These venues exemplify the CTSA Program’s broader commitment to trainee-centered workforce development. The program’s lead founder, Dr. H. William Schnaper, initiated the WSVS Working Group. Following his passing in 2020, the program was named in his honor.

The WSVS program embodies the mission and vision of NCATS by transforming research observations into health solutions and accelerating the development of more treatments for all people [2]. The program reflects NCATS’s core translational science principles, which emphasize cross-disciplinary collaboration, process innovation, boundary-spanning partnerships, and a focus on unmet medical needs while cultivating a diverse and skilled workforce [5]. Achieving this mission depends on educational and career development pathways that prepare a translational science workforce to advance discoveries from the laboratory to the clinic. Here, we describe the evolution of the WSVS program, provide a detailed overview of its structure and activities, and report outcomes from surveys conducted following each mini-symposium. The WSVS program was intentionally designed to serve as a flexible blueprint for other training initiatives in related biomedical workforce development domains.

## Materials and methods

The WSVS mini-symposium provided trainees with a platform to effectively communicate their science through structured presentations and interactive Q&A sessions. The symposium was intentionally designed to promote both scientific and geographic diversity among presenters, highlighting the expansive scientific remit of NCATS and the national reach of the CTSA consortium. Presentation topics spanned the full translational science spectrum – from fundamental animal studies to clinical research and public health implementation – showcasing the breadth of inquiry and innovation among TL1/T32 trainees.

Approximately four months prior to each symposium, the Mini-symposium Program Committee finalized the symposium topic, abstract submission deadline, and event date. Topic selection was guided by group discussions that prioritized translational potential and scientific diversity. Following this, “Save the date” emails were distributed to trainees via an email listserv, maintained by the National TL1/T32 Trainee Representatives, providing details on the session and abstract deadline.

After abstract submission, a panel of at least 12 judges, consisting of both trainees and program directors, was convened maintaining approximately a 2:1 trainee-to-director ratio. Abstract submissions were judged on novelty, relevance to the session theme, clarity, significance, and methodological rigor (Table S1). Scores for each abstract were aggregated, and the top 8–10 submissions were selected for presentation.

Selected trainees were given one week to confirm or decline participation. Once the final presentation list was confirmed, the selections were announced via the T trainee listserv, including each trainee’s name, presentation title, and CTSA hub. The WSVS Working Group then designated two trainees to serve as session moderators. These moderators developed promotional materials, including flyers, which were distributed to T trainees and CTSA T-program administrators for broader dissemination.

One week prior to the symposium, moderators conducted a webinar rehearsal. The goals were to familiarize presenters with the Zoom webinar format and ensure that moderators could effectively manage timing and logistical aspects. Moderators shared a presentation judging rubric with trainees in advance of the mini-symposium (Table S2). The rubric served as an educational tool to guide trainees in the preparation and refinement of their presentations, emphasizing three core domains of effective scientific communication: scientific rigor, engagement, and professionalism. Scientific rigor criteria evaluated the clarity and importance of the research question, methodological rigor, and the strength and interpretation of findings. Engagement criteria assessed clarity and pacing of spoken delivery, logical organization, and effective slide structure. Professionalism criteria evaluated preparedness, appropriate use of time, audio-visual quality in the virtual environment, and overall visual esthetics.

Within one month after the symposium, all presenters and audience members were sent a follow-up survey. The survey assessed trainees’ preparation for their presentations, their perceptions of the symposium’s organization, and whether they would recommend the opportunity to peers. Survey responses for years 2022 and 2023 were not available for analysis; response rates were similar across years. All survey respondents gave informed consent, and the IRB at The University of Rochester approved all research procedures.

## Results

As of December 2025, seven WSVS mini-symposia have been held, beginning with the inaugural event on October 11, 2021. The duration of these symposia has ranged from 90 minutes to 3 hours, with the majority lasting approximately 2 hours (Table 1). In 2021 and 2022, only one mini-symposium was held per year; this number expanded to two events per year starting in 2023.

**Table 1.** WSVS mini-symposia presentations Table 1 long description.

Date	Title and number of participants	Host institution/ moderators	Presenters		Type of CTS topic
Oct 11, 2021 11–2 pm EST	*COVID-19: Innovation and Evolution of Research in the Pandemic*	**Host** *Kelsey Bogue* Executive Dir, UC ITM **Moderators** Laura Magda, MD Maylyn Martinez, MD	***Basic Sciences*** Stephanie Baringer Caitlynn Barrows Louise Helms Aaren Kettelhut, MPH Victoria Morris Raven M. Osborn	***Clinical Sciences*** Jennifer Applebaum, MS & Carlyn Ellison, MPH, CPH Cory Jacinto, MPH Alaa Kassir, MD Tenzin Namdul, PhD	Basic/Translational 3 Clinical Research 5 Implementation Science/Population Health 2
Dec 13 2022 1–3 pm EST	*Circumventing Road Blocks in Clinical and Translational Science*	**Host** *Kathryn Sandberg, PhD* TL1 Director, GHUCCTS **Moderators** *Session 1* Alex Brunfeldt, PhD Tara Jenson *Session 2* Rosemarie Murray Albert Holler	***Session 1*** Sara Bybee, PhD, LCSW Rachel E. Bishop Carly Herbert Marissa LoCastro Stephanie Zawada M.S. Aarti C. Bhat, MS	***Session 2*** Pryce Michener, MS Deborah George, MS Alexander Andersohn Jessica Herstine, MS Heather Holman	Basic/Translational 2 Clinical Research 5 Implementation Science/Population Health 4
Apr 25, 2023 11:30–1 pm EST	*Innovations in Health*	**Host** *Jay F. Piccirillo, MD* TL1 Director, WashU ITHS **Moderators** Pat Kung, PhD Edwin Baldelomar, PhD	***Speakers*** Donald Bourne Mohamed Addani Deborah George, MS Marrisa Therriault		Basic/Translational 1 Clinical Research 2 Implementation Science/Population Health 1
Dec 12, 2023 1–3 pm EST	*Application of Emerging Technologies in Translational Science*	**Host** *Dan Moglen, PhD* Chair, WSVS Program Committee UC Davis, CTSC **Moderators** Baqir Kewdai Desiree Sigala, PhD Research Admin Fellow, CTSC	***Session 1*** Adam Kaminski Zeeba Manavi, MS Deborah George, MS Casiana Gonzales	***Session 2*** Juliet Strauss Morgan Manweiler, MEd; Eds Justin James Fitzgerald Casiana Gonzales	Basic/Translational 4 Clinical Research 4
Mar 27, 2024 12–2 pm EST	*Crossing Boundaries in Clinical and Translational Science*	**Hosts** Patrick McDeed *Dexter L. Lee, PhD TL1 Co-Director, GHUCCTS* **Moderators** *Session 1* Angelica Medina, PhD Rong Xin Liu *Session 2* Brox Pagni, PhD Edward Huang	***Session 1*** Dana Julian Richard Garner Elizabeth Cervantes Rebecca Horowitz Angelica Medina, PhD	***Session 2*** Aniruddh Ajith Darian Williams, PhD Marissa Behun Grace Kim Brox Pagni, PhD	Basic/Translational 3 Clinical Research 6 Implementation Science/Population Health 1
Dec 11, 2024 12–2 pm EST	*Focusing on unmet needs in Clinical and Translational Science*	**Host** *Mark Hatcher, GHUCCTS* **Moderators** *Session 1* Emily Stahl Alyssa Beck *Session 2* Natalie Deana Badillo Hayley Chang	***Session 1*** Hridya Rao Gardner Haley Bergman Najjuwah Walden Gyutae Lim, PhD Edwin Omar Rivera-Lopez	***Session 2*** Mary Bucklin Rojina Nekoonam Ryan Staples, PhD Anna Vaeth Brittany Martinez, PhD	Basic/Translational 3 Clinical Research 7
Dec 10, 2025 12–2 pm EST	Breaking Bottlenecks: Innovative Strategies in Clinical Translational Science	**Host** *Zeeba Manavi*, GHUCCTS **Moderators** *Session 1* Sawson Salah Rebecca Singer Cohen *Session 2* Brittany M. Hufft-Martinez, PhD Jason Hatfield	***Session 1*** Matthew W. Liao Erin Markle Akhi Palleria Ben Clements, PhD	***Session 2*** Natalie West Gemalene Sunga Caron Gundlach Cailityn A. Thomas, PhD	Basic/Translational 5 Clinical Research 3

CCaTS = Center for Clinical and Translational Science; CCTS = Center for Clinical and Translational Science; CTSC = Clinical Translational Science Center; CTSI = Clinical Translational Science Institute; CTRI = Clinical Translational Research Institute; GHUCCTS = Georgetown Howard Universities Center for Clinical and Translational Science; ICTR = Institute for Clinical and Translational Research; ITM = Institute for Translational Medicine; ITS = Institute for Translational Sciences; KU = University of Kansas; Penn State = Pennsylvania State University; Pitt = University of Pittsburgh; SC = South Carolina; UAB = University of Alabama at Birmingham; UC Davis = University of California at Davis; UC = University of Chicago; UCLA = University of California at Los Angeles; UF = University of Florida at Gainesville; UK = University of Kentucky; UMass = University of Massachusetts; UR = University of Rochester; UT = University of Texas; UTMB = University of Texas Medical Branch; Utah = University of Utah.

**Figure 1.**
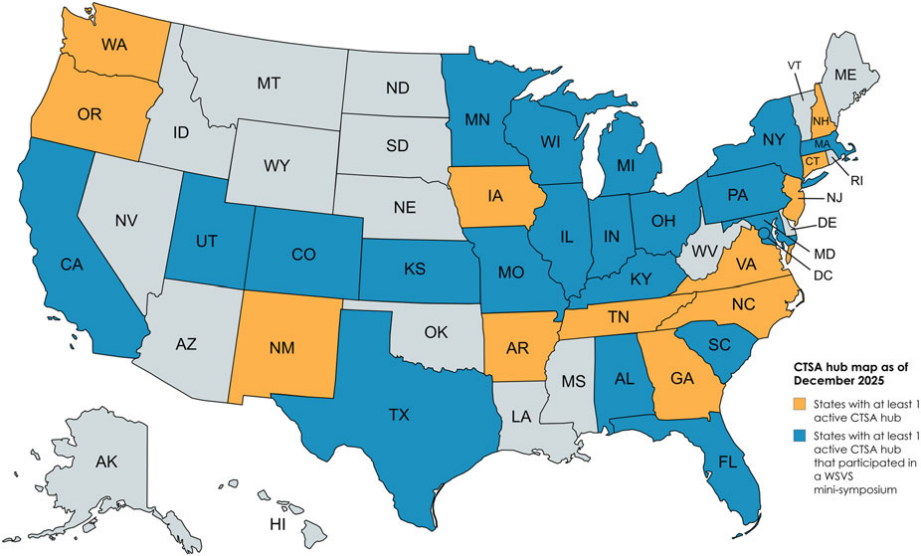
Geographic distribution of CTSA hubs. Of the 33 states and districts with CTSA hubs, 21 states have contributed at least one presenter or moderator (blue). Map created with mychart.net. Figure 1 long description.

Participation has been robust: 29 CTSA hubs have contributed a speaker, moderator and/or host. Across seven events, 62 speakers from 28 CTSA hubs have presented, with an average number of 8 CTSAs represented per symposium (Table 1). Figure 1 illustrates the geographic distribution of participating CTSA hubs. Of the 33 states and districts with CTSA hubs, 21 have contributed at least one presenter or moderator. Fifteen of the participating hubs contributed to more than one symposium; among these, eight participated twice, while the other seven participated three to five times. In 2024, trainees from the NCATS intramural training program also served as speakers and moderators at both the fall and spring symposia. High participation rates were facilitated by a centralized trainee email listserv, curated and updated by the National TL1/T32 Trainee Representatives. Three times per year, these representatives attended the National TL1/T32 Directors’ Meeting to encourage program directors and administrators to update trainee contact information. As of Q4 2025, the listserv contains contact details for 430 trainees. The listserv reaches approximately 85% of listed addresses; the remaining 15% are undeliverable or removal requests from trainees. Of the 47 CTSA T programs nationwide, 38 consistently update their trainee information, reinforcing the listserv as a reliable communication and recruitment tool.

**Figure 2.**
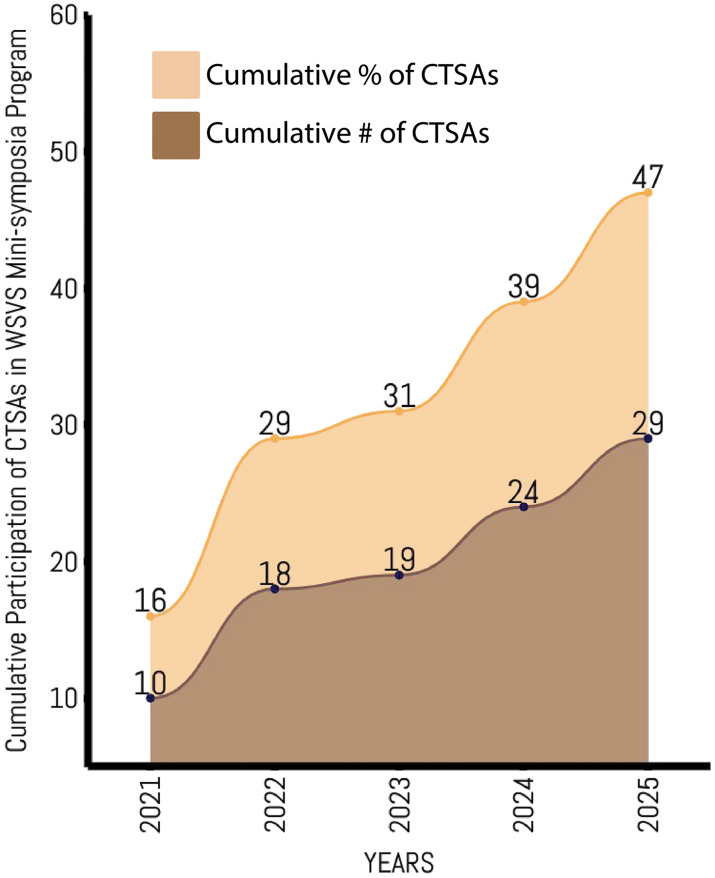
Cumulative participation of CTSAs in the WSVS mini-symposium program since its initiation. The growing number (brown) and percentage (tan) of CTSAs that have participated in the WSVS mini-symposium program since its inception in 2021 through December 2025. Figure 2 long description.

Figure 2 illustrates the growth in mini-symposium participation from the inaugural mini-symposium in October 2021 through December 2025. Each year, between one and eight new CTSA hubs have contributed at least one trainee presenter or moderator.

Regarding trainee demographics, 79% (49/62) of speakers were predoctoral students while 21% (13) were postdoctoral fellows. All mini-symposia included trainee moderators with 63% (14/22) predoctoral and 36% (8/22) postdoctoral participants (Table 1). Hosts for the first four symposia were experienced members of their CTSA hub; however, in 2024 predoctoral students assumed hosting roles for both spring and fall events. The research presented spans the full spectrum of translational science: basic/preclinical (34%; 21/61), clinical (52%; 32/61), clinical implementation and public health (13%; 8/61) (Table 1). While basic/preclinical research has predominated, it remains to be determined whether this distribution reflects the broader trends across TL1/T32 programs nationwide.

Survey data from 4 mini-symposia (missing 2022 and 2023 data) generated 21 presenter respondents (*N* = 13 female/woman, *N* = 6 male/man, *N* = 1 gender queer, *N* = 1 non-binary) with 15% (3/21) identifying as Hispanic and 85% (18/21) as non-Hispanic. Most of the respondents were predoctoral (*N* = 17): 11 pursuing their PhD, 5 their MD-PhD, and 1 their MD. Four respondents were postdoctoral. The majority of respondents (*N* = 20/21, 95%) “Agreed” or “Strongly Agreed” that presenting at the symposium was valuable in terms of advancing their research. Similarly, all respondents (*N* = 21/21, 100%) “Agreed” or “Strongly Agreed” that presenting at the symposium was valuable in terms of their professional development. Several respondents (*N* = 11/21, 52%) felt that they had received valuable feedback from the attendees and that they met at least one person whom they are likely to contact in the future (*N* = 10/21, 48%). All respondents (*N* = 20/20, 100%) felt that the operational logistics were well executed and most (*N* = 19/21, 90%) would recommend that other trainees present their research at the symposium. The survey concluded with open-ended questions. In response to: What aspects of your experience did you find most useful? One trainee wrote:

*Being able to present my work to scholars from diverse backgrounds, as well as to learn from other presenters. Coming from a social science field, it was also a great learning experience to see how researchers in biomedical field present their work.*

A total of 61 survey responses were collected from symposium attendees. Most attendees were either predoctoral (*N* = 28) or postdoctoral trainees (*N* = 14). Professors, Associate and Assistant Professors made up nearly a fifth of attendees (N = 13) and the remaining were staff (*N* = 4) and undergraduate students (*N* = 2). Several respondents (*N* = 40/51, 78%) “Agreed” or “Strongly Agreed” that attending the symposium was a valuable experience. Similarly, a large portion of respondents (*N* = 47/53, 89%) “Agreed” or “Strongly Agreed” that they would recommend attending a TL1/T32 symposium to a colleague. Most respondents “Agreed” or “Strongly Agreed” (*N* = 48/53, 91%) that the operational logistics were well executed and that the time allocated for each trainee presentation (*N* = 50/59, 85%) and the overall symposium (*N* = 49/61, 80%) was just right. Attendees were also asked to describe what they found most useful about the mini-symposium; one attendee wrote:

*I appreciated viewing and learning about the broad spectrum of research topic areas, as well as seeing each presenter’s style of communication and slide presentations.*

## Discussion

The WSVS Mini-symposium was developed to create an accessible, inclusive, and sustainable forum for TL1/T32 trainees to share their research and strengthen communication and leadership skills across the translational spectrum. Our findings demonstrate that a virtual, trainee-centered format can effectively engage participants from diverse institutions and disciplines while fostering national collaboration. Virtual platforms have become essential tools for advancing equity in scientific training, particularly for predoctoral and postdoctoral researchers with limited financial or logistical resources. Prior studies have shown that online and hybrid symposia can broaden participation [6, 7] and improve the financial and environmental sustainability of scientific meetings [8].

### Engagement trends and future outreach

Most speakers in the WSVS Mini-symposium series have been predoctoral students rather than postdoctoral fellows. This pattern likely reflects the composition of CTSA training programs, which include a greater proportion of predoctoral (62.5%) compared to postdoctoral (37.5%) trainees [9]. Additionally, predoctoral students may perceive a stronger need to practice scientific communication, which may further explain their predominance among presenters. Approximately half of the CTSAs with TL1/T32 training programs have participated in at least one WSVS Mini-symposium. Of these, over half have participated in multiple events, suggesting that both program directors and trainees recognize the value of the series. The type of research pursued by the trainees presenting at the Mini-symposia is reflected in the research focus of the majority of TL1/T32 programs. Based on a survey conducted in 2019, the majority of TL1/T32 programs encompassed basic/preclinical research and various degrees of clinical implementation and public health [9]. Together, the steady growth in new CTSA hub participation and the breadth of research represented underscore the program’s expanding visibility and appeal.

To broaden awareness and avoid limiting participation across the CTSA consortium, future outreach efforts should expand current promotional strategies by pairing WSVS program advertising with T-centric events at the annual Translational Science meeting, including the three-minute thesis competition, trainee luncheon, and mock study section. Enhanced use of the national trainee listserv would also facilitate promotion of the mini-symposia. However, maintaining this listserv remains an ongoing challenge, as approximately 15% of trainee emails were returned as undeliverable in July 2025. Improving listserv accuracy and dissemination processes will be critical for sustaining engagement. Although we have not yet conducted a survey of non-participating sites, we speculate that limited awareness of the program may contribute to lack of participation by some CTSA training programs.

### Strategic alignment with national meetings

Starting in 2022, the mini-symposium was promoted as a forum for trainees to prepare for the annual Association for Clinical and Translational Science meeting: *Translational Science*. Symposia were scheduled before *Translational Science* to allow trainees to rehearse their presentations. To further encourage participation, abstracts were due after the *Translational Science* abstract deadline, and trainees were invited to submit the same abstracts they had prepared for *Translational Science*. This strategy has likely contributed to a fourfold increase in abstract submissions over the past six mini-symposia. Another possible contributing factor is promotion by the increasing number of trainees who have participated in prior mini-symposia. Only 10% of trainees submitting abstracts to *Translational* Science are selected for platform presentations, which is similar to percentages at other national scientific meetings. Surveys indicate mini-symposia participants appreciated the opportunity to present their findings and respond to questions from a diverse audience including those outside their specific discipline. Applicants who were not selected for platform presentations at *Translational Science* had the opportunity to serve as moderators or judges, with nearly 90% agreeing to participate. These roles were highly valued by trainees, both for the career-building opportunity at a national meeting and for the targeted coaching they received in session moderation and evaluation of oral presentations – core professional skills rarely emphasized in traditional scientific training programs. Collectively, these experiences may foster self-confidence and encourage trainees to pursue future leadership roles within their respective disciplines.

Improvements in participation have been driven in part by consistent updates to the WSVS trainee listserv. Structured, repeated reminders during meetings and via email to CTSA program directors and administrators have enhanced the accuracy and completeness of trainee contact information. To build on this progress, the WSVS working group recommends integrating contact information verification into trainee onboarding and CTSA grant renewal procedures. Embedding listserv maintenance into existing administrative workflows will provide a more systematic, sustainable approach to ensuring long-term engagement and communication.

Compiling a parallel list of CTSA administrative staff responsible for trainee program coordination will further improve communication with CTSA hubs. These administrative contacts are often more accessible than T directors for day-to-day tasks and typically maintain up-to-date trainee records, positioning them to support timely listserv updates. Clear communication with these administrators will underscore the importance of promptly updating trainee information following staff or trainee changes, thereby supporting sustained outreach and consistent engagement with national WSVS initiatives.

Establishing an alumni list to maintain contact with former trainees after program completion is another valuable strategy. Program administrators could be encouraged to collect non-institutional email addresses from trainees prior to graduation to support continued communication. This alumni network would facilitate ongoing engagement through post-training surveys, invitations to serve as guest speakers at future events, success story highlights, and opportunities for graduates to reflect on how the TL1/T32 program influenced their career trajectory. Maintaining these connections would help reinforce the translational training pipeline and provide a mechanism for evaluating the long-term impact of the WSVS program.

### Empowering trainees through dedicated learning forums

Trainee-led symposia serve as dynamic platforms for developing a broad range of professional skills essential for success in scientific careers. By organizing calls for abstracts, recruiting judges and moderators, and managing logistics, trainees gain firsthand experience in event planning that mirrors the structure of larger scientific conferences. Participation in symposium committees further cultivates trainees’ evaluative and organizational skills, as they contribute to session programming, abstract selection, and presentation coordination. Moderating sessions also equip trainees with practical abilities in time management and audience engagement by guiding speakers and facilitating Q&A discussions.

In addition to skill development, trainee-led symposia foster professional networking and mentorship opportunities [10, 11]. These events offer structured yet accessible environments for early-career researchers to connect with peers and near-peers through collaborative committee work and judging activities. Such interactions often lead to lasting relationships and future collaborations. Presenting and moderating sessions enhances trainee visibility and credibility within their scientific communities, while informal interactions during Q&A sessions further promote the exchange of ideas and sense of support among emerging trainees. Together, these experiences strengthen professional identity and prepare trainees for active leadership roles in discipline-specific forums.

### Implications for biomedical workforce development

A central goal of the WSVS mini-symposium is to encourage trainees to develop compelling and well-structured presentations tailored to a broad audience. Target audiences range from early-stage predoctoral students to advanced postdoctoral fellows spanning disciplines from basic science to public health. Trainees are also expected to navigate Q&A sessions with professionalism by responding to challenging questions calmy, acknowledging knowledge gaps, and situating their work within broader translational contexts. These experiences not only enhance trainees’ ability to communicate their research effectively but also help shape their scientific identity and presence. Notably, strong presenters often leave lasting impressions and are invited for future speaking engagements, highlighting how clear communication and professional conduct contribute to credibility and a recognizable presence in the field.

In addition to enhancing communication skills, symposia offer invaluable opportunities for professional networking and career advancement. Evidence from in-person conferences highlights this impact: in a large cross-sectional study of German predoctoral and postdoctoral trainees, 18% of predocs and 33% of postdocs reported receiving invitations to present at other institutions following participation in an international conference, while 14% and 27%, respectively, initiated new research collaborations [12]. These findings underscore the tangible career benefits of real-time scholarly exchange and increased visibility. Although virtual formats may not fully replicate the informal interactions of in-person events, they provide scalable and accessible entry points into professional networks that are particularly valuable for early-career scientists with limited travel resources. As virtual and hybrid symposia continue to evolve, they offer significant opportunities to expand equitable participation while fostering connections that can catalyze future collaborations.

### Opportunities for program improvement

Analysis of trainee feedback identified three opportunities to enhance the mini-symposium experience. First, increasing the educational value of presentations by providing structured feedback from judges to trainees using standardized forms that capture both strengths and areas for improvement. Moderators can further strengthen this mechanism by facilitating form completion and ensuring comments are relevant before distribution to trainees. Second, supporting moderator development by soliciting feedback from moderators to assess their preparedness, identify training needs, and understand how the experience influences future leadership engagement at scientific meetings. Finally, refining the abstract and presentation scoring rubrics by gathering feedback on them to maximize their educational impact.

### Conclusion

The WSVS program demonstrates how virtual, trainee-centered learning forums can effectively foster skill development across the translational science spectrum. By eliminating logistical barriers and emphasizing inclusivity, these symposia empower trainees to grow professionally through structured presentations, audience engagement, and peer-led organization and administration. These initiatives directly support NCATS’s mission to build a diverse, multidisciplinary translational workforce, and to promote team science, collaboration, and community engagement. As the biomedical research community increasingly adopts hybrid and virtual approaches to education and training, the WSVS model offers a scalable, adaptable framework that can serve as a blueprint for similar efforts across scientific disciplines and institutional programs.

## Supporting information

10.1017/cts.2026.10802.sm001 Brunfeldt et al. supplementary material Brunfeldt et al. supplementary material

## Supplementary material

The supplementary material for this article can be found at https://doi.org/10.1017/cts.2026.10802.

## Table 1. Long description

Abbrev.: CCaTS, Center for Clinical and Translational Science; CCTS; Center for Clinical and Translational Science; CTSC, Clinical Translational Science Center; CTSI; Clinical Translational Science Institute; CTRI, Clinical Translational Research Institute; GHUCCTS, Georgetown Howard Universities Center for Clinical and Translational Science; ICTR, Institute for Clinical and Translational Research; ITM, Institute for Translational Medicine; ITS, Institute for Translational Sciences; KU, University of Kansas; Penn State, Pennsylvania State University; Pitt, University of Pittsburgh; SC, South Carolina; UAB, University of Alabama at Birmingham; UC Davis, University of California at Davis; UC, University of Chicago; UCLA, University of California at Los Angeles; UF, University of Florida at Gainesville; UK, University of Kentucky; UMass, University of Massachusetts; UR, University of Rochester; UT, University of Texas; UTMB, University of Texas Medical Branch; Utah, University of Utah

Navigate back to Table 1..

## Figure 1. Long description

Geographic distribution of CTSA hubs. Of the 33 states and districts with CTSA hubs, 21 states have contributed at least one presenter or moderator (blue).

Navigate back to Figure 1..

## Figure 2. Long description

Cumulative participation of CTSAs in the WSVS mini-symposium program since its initiation. The growing number (brown) and percentage (tan) of CTSAs that have participated in the WSVS mini-symposium program since its inception in 2021 through December 2025.

Navigate back to Figure 2..
