# Supplementary material for: NCATS TL1/T32 William Schnaper Visiting Scientist program: Trainee-driven mini-symposium
Source: J Clin Transl Sci. 2026 Jul 21;10(1):e140. doi: 10.1017/cts.2026.10802 (PMC13458299; doi:10.1017/cts.2026.10802)
Supplement: Supplementary file 1 [file S2059866126108024sup001.docx]

**SUPPLEMENT**

Table S1: Abstract judging rubric

| **Instructions for Abstract review:** Please read each abstract carefully. Based on the criteria below, s*core abstracts with an overall score of 1, 2, or 3. Each abstract should receive only one score. Make your best judgement call for abstracts that do not perfectly fit into a score of 1, 2, or 3.* | | | |
| --- | --- | --- | --- |
|  | **Score** | | |
| **Criteria** | **1** | **2** | **3** |
| **Novelty** | Highly Unique | Some Originality | None to Very little Originality |
| **Relevance to Theme** Example: *"Application of Emerging Technologies in Translational Science"* | High Relevance | Some Relevance | Little to No Relevance |
| **Clarity** | Highly Organized and Clear | Somewhat organized and clear | Not clear or organized |
| **Significance/Impact** | Highly significant and impactful to the field | Somewhat significant and impactful to the field | Not significant or impactful to the field |
| **Methodology** | Clear relevant hypothesis which methodology accurately tests. Methodology well executed. | Clear hypothesis. Methodology relevant with only minor weaknesses in design | Hypothesis unclear. Methodology doesn’t really test hypothesis or poorly conducted. |

Table S2: Presentation judging rubric

| **Instructions for Presentation Judging Criteria:** Based on the criteria below, score each presentation with an overall score of 1-9, 9 is best. | | | | | | | | | |
| --- | --- | --- | --- | --- | --- | --- | --- | --- | --- |
|  | **Overall Score** | | | | | | | | |
| **Criteria** | **1** | **2** | **3** | **4** | **5** | **6** | **7** | **8** | **9** |
| **Scientific Merit**   - High translational potential - Innovative techniques - Rigorous approach/methods - Clear interpretation of results - Awareness of limitation or future research needs | Very poor | Poor | Moderately Below Average | Slightly Below Average | Average | Slightly Above Average | Moderately Above Average | Excellent | Exceptional |
| **Engagement & Communication**   - Speaking is clear, well-paced, with no jargon - Clear logic order and easy to follow slides - Appropriate use of time - Clear preparation for questions |  |  |  |  |  |  |  |  |  |
| **Professionalism**   - Plain Zoom background and good lighting with working slides, video, and audio - Appropriate use of animations that are visually appealing with uncluttered text, graphics, slides, backgrounds, etc. |  |  |  |  |  |  |  |  |  |
